# Neuroprotective and Cognitive-Enhancing Effects of Microencapsulation of Mulberry Fruit Extract in Animal Model of Menopausal Women with Metabolic Syndrome

**DOI:** 10.1155/2017/2962316

**Published:** 2017-10-12

**Authors:** Supannika Kawvised, Jintanaporn Wattanathorn, Wipawee Thukham-mee

**Affiliations:** ^1^Department of Physiology and Graduate School (Neuroscience Program), Faculty of Medicine, Khon Kaen University, Khon Kaen 40002, Thailand; ^2^Integrative Complementary Alternative Medicine Research and Development Center, Khon Kaen University, Khon Kaen 40002, Thailand; ^3^Department of Physiology, Faculty of Medicine, Khon Kaen University, Khon Kaen 40002, Thailand

## Abstract

Currently, the neuroprotectant and memory-enhancing agent for menopausal women with metabolic syndrome is required. Based on the advantages of polyphenolics on numerous changes observed in menopause with metabolic syndrome and the encapsulation method, we hypothesized that microencapsulated mulberry fruit extract (MME) could protect brain damage and improve memory impairment in an animal model of menopause with metabolic syndrome. To test this hypothesis, MME at doses of 10, 50, and 250 mg/kg was given to female Wistar rats which were induced experimental menopause with metabolic syndrome by bilateral ovariectomy (OVX) and fed with high-carbohydrate high-fat (HCHF) diet for 8 weeks. Spatial memory together with neuron density, oxidative stress status, acetylcholinesterase, and phosphorylation of Erk in the hippocampus was assessed at the end of the study. It was found that MME decreased memory impairment, oxidative stress status, and AChE activity but increased neuron density and Erk phosphorylation in the hippocampus. Therefore, the neuroprotective and memory-enhancing effects of MME might partly involve the enhanced cholinergic function and Erk phosphorylation but decreased oxidative stress status in hippocampus. Therefore, MME is the potential novel neuroprotectant and memory-enhancing agent for menopause with metabolic syndrome. However, further research especially clinical trial is still necessary.

## 1. Introduction

The number of menopausal women is continually increasing worldwide [1]. Recently, it has been demonstrated that the prevalence of chronic diseases including metabolic syndrome in menopausal women is increased [2, 3]. Moreover, both menopausal condition and metabolic syndrome can induce memory impairment and decrease hippocampal plasticity [4–7]. Based on the rising trends of both menopause and metabolic syndrome mentioned earlier, the memory impairment in menopausal women with metabolic syndrome is increasing its importance and is recognized as one of the important health problems. Unfortunately, less information concerning this issue is available. Moreover, the current therapeutic strategy is still not in satisfaction level. Therefore, this problem should be concerned and the successful strategies for combating this condition are required.

Recently, it has been demonstrated that the substances which are rich in polyphenolic compounds especially anthocyanins can improve both metabolic syndrome and cognitive impairment [8, 9]. Since polyphenolic compounds including anthocyanins have low stability toward environment condition during processing and storage, it is a hard task force to incorporate the mentioned substance into foods and health products. However, encapsulation has been reported to be an effective way to introduce such substances into the mentioned products. Encapsulating agent can be served as a protector coat against adverse environmental condition resulting in the increased stability. In addition, encapsulated compounds are also easier to handle.

Ripen fruits of mulberry or *Morus alba* (Moraceae family) are rich in polyphenolic compounds especially anthocyanins. The mulberry fruit extract exerts the neuroprotective and memory-enhancing effects in an animal model of vascular dementia [10]. Since the neurodegeneration and memory impairment both in vascular dementia [10] and in a menopausal woman with metabolic syndrome [11] are associated with oxidative stress, the neuroprotective and cognitive-enhancing effects of mulberry extract have been focused. Based on the benefits of anthocyanins and mulberry extract together with the encapsulation advantage mentioned earlier, we hypothesized that the encapsulated mulberry extract could improve hippocampal damage and memory impairment in postmenopausal metabolic syndrome. To elucidate this issue, this study was carried out to determine the neuroprotective and cognitive-enhancing effects of encapsulated mulberry extract in an animal model of postmenopausal metabolic syndrome induced by high-carbohydrate high-fat diet.

## 2. Materials and Methods

### 2.1. Preparation of Mulberry Fruit Extract

Ripen mulberry fruits (*Morus alba* L.) were collected from the Queen Sirikit Department of Sericulture Center, Udon Thani Province. The fresh mulberry fruits were cleaned and dried with an oven (Memmert GmbH, USA) at 60°C for 72 hours. The dried mulberry was grounded to fine powder and extracted by maceration technique with 50% hydro-alcoholic (1% *w*/*v*) for 24 h and then filtered with Whatman number 1 filter paper. All yielded extracts were dried with the oven (Memmert GmbH, USA) at 60°C for 24 hours and kept at 4°C until used.

### 2.2. Preparation of Microencapsulated Mulberry Extract

Maltodextrin dextrose equivalent 10 (DE10) was selected as encapsulation matrix. It was mixed with mulberry extract at the ratio of 9 : 1 (*w*/*w*). The mixture was dissolved in warm distilled water at 50°C and stirred for 30 minutes. The solution was frozen at −20°C for 18 hours in a freezer and subjected to drying in a freeze-dryer (Labconco freeze dryer, Labconco Corporation, Kansas City, MO, USA) for 48 hours (−86°C, 0.008 mbar). The dry sample was packaged and stored in a desiccator containing silica gel at 4°C.

### 2.3. Measurement of Total Phenolic Compound Contents

The total phenolic compound content of the encapsulated mulberry extract was determined by using the Folin-Ciocalteu colorimetric method in microplate reader (iMark™ Microplate Absorbance Reader) [12, 13]. A 20 *μ*l of the extract was mixed with 158 *μ*l of distilled water and 20 *μ*l of 50% *v*/*v* Folin-Ciocalteu reagent (Sigma-Aldrich, USA) which was freshly prepared. The mixture was incubated for 8 minutes. Then, 30 *μ*l of 20% Na_2_CO_3_ (Sigma-Aldrich, USA) was added and subjected to a 2-hour incubation period at room temperature in a dark room. The absorbance was measured at 765 nm. Result was expressed as mg gallic acid equivalent (GAE)/mg microencapsulated mulberry extract. Various concentrations of gallic acid (Sigma-Aldrich, USA) were used as a standard calibration curve.

### 2.4. Measurement of Flavonoid Content

The determination of flavonoid content was performed by aluminum chloride method [14]. This process was based on the formation of aluminum-flavonoid complexes. All assessments were performed in triplicate. In brief, 100 *μ*l of the extract at various concentrations was mixed with 100 *μ*l of 2% methanolic aluminum chloride (Sigma-Aldrich, USA) and subjected to the 30-minute incubation period at room temperature in dark room. Then, the absorbance at 415 nm was taken against the suitable blank. Various concentrations of quercetin (Sigma-Aldrich, USA) were used for the standard calibration curve preparation. Results were expressed as *μ*g quercetin equivalent/mg microencapsulated mulberry extract.

### 2.5. Determination of Free Radical Scavenging Activities

The scavenging activity against free radicals of the encapsulated mulberry extract was assessed via 1,1-diphenyl-2-picryl-hydrazyl (DPPH), ferric reducing antioxidant power (FRAP), and 2,2′-azinobis-3-ethylbenzothiazoline-6-sulphonic acid (ABTS) assays. For DPPH assay, an aliquot of 0.15 mM DPPH in methanol (180 *μ*l) (Sigma-Aldrich, USA) was mixed with 20 *μ*l of various concentrations of encapsulated mulberry extract and incubated for 30 minutes. The absorbance was measured against blank at 517 nm via microplate reader (iMark Microplate Absorbance Reader) [15, 16]. Ascorbic acid was used as positive control. Results were expressed in terms of EC_50_ (concentration in micrograms per milliliter required to inhibit DPPH radical formation by 50%).

The determination of antioxidant activity via FRAP assay is based on the ability of the tested substance to convert ferric tripyridyltriazine (Fe^3+^-TPTZ) to ferrous tripyridyltriazine (Fe^2+^-TPTZ). FRAP working solution containing 10 mM TPTZ (Sigma-Aldrich, USA), 20 mM ferric chloride solution (FeCl_3_) (Sigma-Aldrich, USA), and 300 mM acetate buffer (Sigma-Aldrich, USA) at the volume of 190 *μ*l was mixed with 10 *μ*l of encapsulated mulberry extract. After a 10-minute incubation at 37°C, the solution was determined for the absorbance against blank at 593 nm [17]. Ascorbic was used as positive control, and results were expressed as EC_50_ value.

The 2,2′-azinobis-3-ethylbenzothiazoline-6-sulfonic acid (ABTS) was also used to determine the free radical-scavenging activity of the encapsulated mulberry extract [18]. In brief, various concentrations of the encapsulated mulberry extract (30 *μ*l) were reacted with the stock solution containing 7 mM ABTS (Sigma-Aldrich, USA) and 2.45 mM potassium persulfate (Sigma-Aldrich, USA; freshly prepared). The absorbance was measured at 734 nm.

The antioxidant activity of each sample was expressed in terms of EC_50_ (concentration in micrograms per milliliter required to inhibit ABTS radical formation by 50%) calculated from the log-dose inhibition curve.

### 2.6. Measurement of Acetylcholinesterase Inhibitory (AChEI) Activity

AChE suppression activity of the encapsulated mulberry was determined by colorimetric method according to the method of Elmann et al. [19]. This method is based on the determination of a yellow color of 5,5′-dithiobis (2-nitrobenzoic acid) produced by the hydrolysis of acetylcholine by acetylcholinesterase (AChE). In brief, various concentrations of encapsulated mulberry extract at the volume of 25 *μ*l each were added to the reaction mixture containing 50 *μ*l of Tris-HCl (50 mM, pH 8.0) (Sigma-Aldrich, USA), 75 *μ*l of 3 mM 5,5′-dithio-bis-2-nitrobenzoic acid (DTNB) (Sigma-Aldrich, USA), and 25 *μ*l of 15 mM thiocholine iodide (ATCI) (Sigma-Aldrich, USA) and 25 *μ*l of AChE (0.22 U/ml) (Sigma-Aldrich, USA). After mixing, the reaction mixture was incubated at room temperature for 5 minutes, and the absorbance at 412 nm was recorded with microplate reader (iMark Microplate Absorbance Reader). Percentage of inhibition was calculated by comparing the rate of enzymatic hydrolysis of ATCI for the samples to that of the blank (50% aqueous methanol in buffer). Donepezil (1–32 mM) (ARICEPT®, USA) was used as a reference standard. The AChE inhibition activity of each sample was expressed in terms of EC_50_. Every sample was assessed in triplicate.

### 2.7. Finger Print Chromatogram Assessment

The phenolic profiles of mulberry extract and encapsulated mulberry extract consisting of cyanidin-3-glucoside (Sigma-Aldrich, USA), gallic acid (Sigma-Aldrich, USA), and quercetin-3-O-rutinoside (Sigma-Aldrich, USA) were determined by high-performance liquid chromatography (HPLC). Chromatography was performed by using a Waters® system equipped with a Waters 2998 photodiode array detector. Chromatographic separation was performed using Purospher® STAR, C-18 encapped (5 *μ*m), LiChroCART® 250-4.6, and HPLC-Cartridge, Sorbet Lot number HX255346 (Merk, Germany). The mobile phase (HPLC grade) consisted of 100% methanol (solvent A) (Fisher Scientific, USA) and 2.5% acetic acid (solvent B) (Fisher Scientific, USA) in deionized (DI) water was used to induce gradient elution. The gradient elution was carried out at a flow rate of 1.0 ml/min with the following gradient: 0–17 min, 70% A; 18–20 min, 100% A; and 20.5–25 min, 10% A. The sample was filtered (0.45 *μ*m, Millipore), and a direct injection of tested sample at the volume of 20 *μ*l on the column was performed. The chromatograms were recorded at 280 nm using UV detector, and data analysis was performed using EmpowerTM3.

### 2.8. Experimental Protocol

Female Wistar rats (weighing 200–250 g, 10 weeks old) were obtained from National Laboratory Animal Center, Salaya, Nakhon Pathom, Thailand. The rats were kept under standard laboratory conditions with food and water ad libitum and housed in standard metal cages (5 per cage). Temperature was controlled at 23 ± 2°C on 12 : 12 h light-dark cycle. All procedures and experimental protocols were approved by the Institutional Animal Ethics Committee of Khon Kaen University (Record number AEKKU 27/2017). After 1 week of acclimatization, the animals were divided into 7 groups as follows:

- Group I (normal diet (ND) + vehicle): all rats in this group received normal diet (4.5% fat, 42% carbohydrate, and 24%protein) and were treated with vehicle.
- Group II (HCHF + vehicle): all animals in this group received HCHF diet and treated with vehicle.
- Group III (OVX-HCHF diet + vehicle): all rats in this group were subjected to bilateral ovariectomy (OVX), received HCHF diet, and treated with vehicle.
- Group IV (OVX-HCHF diet + isoflavone): rats had been subjected to bilateral ovariectomy, fed with HCHF diet, and treated with isoflavone at a dose of 15 mg/kg BW.
- Group V-VII (OVX-HCHF diet + microencapsulated mulberry extract (MME)): all rats in these groups were subjected to OVX, received HCHF diet, and treated with MME at various doses ranging from 10, 50, and 250 mg/kg BW.

In this study, all OVX rats were anesthetized with thiopental sodium at a dose of 40 mg/kg BW prior to the induction of experimental menopause by bilateral ovariectomy. After 1 week of operation, OVX rats in group III–group VII were fed with high-carbohydrate high-fat diet (HCHF; 35.83% fat, 35.54% carbohydrate, and 28.63% protein) in order to induce metabolic syndrome. After 20 weeks of the feeding period, rats which showed percent change of body weight more than 25 percent, the homeostasis model assessment-estimated insulin resistance (HOMA-IR) index and plasma angiotensin-converting enzyme levels higher than the control group were selected for further study. Then, the recruited animals were randomly assigned for the interventions including vehicle, isoflavone, and MME at various doses ranging from 10, 50, and 250 mg/kg BW. All animals were assessed for spatial memory after the single dose of treatment and at the end of 8-week intervention period. In addition, neuron density, the oxidative stress status, AChE, and the expression of Erk signal transduction in the hippocampus were also investigated at the end of the study.

### 2.9. Morris Water Maze Test

The water maze is a metal circle pool at diameter of 147 cm and 60 cm in depth, and water (25 ± 1°C) was filled with white nontoxic milk powder to a depth of 40 cm. It was divided into 4 equal quadrants (Northeast, Southeast, Southwest, and Northwest) by 2 imaginary lines crossing the center of the pool. A removable platform was immersed in the center of one quadrant. Each animal was trained to memorize the location of the platform by forming the association information between its location and the location of platform by using external cues. After 4 training sessions, the animals were determined the time which required for finding the platform and climbed onto the platform or escape latency. The retention time was also determined by exposing the animals to the same condition except that the platform was removed from the location previously immersed. In this case, the time which the animals spend swimming in the quadrant which previously contained the platform was regarded as retention time that was recorded.

### 2.10. Histological Procedure and Nissl Staining

The brains were perfused transcardially with fixative solution containing 4% paraformaldehyde (Sigma-Aldrich, USA) in 0.1 M phosphate buffer pH 7.4 overnight at 4°C. Then, they were infiltrated with 30% sucrose (Merck, Germany) solution for 48–72 h. Serial sections of tissues were cut frozen on cryostat (Thermo Scientific™ HM 525 Cryostat) at 10 *μ*m thick. All sections were picked up on slides coated with 0.3% aqueous solution of gelatin containing 0.05% aluminum potassium sulfate (Sigma-Aldrich, USA). The triplicate coronal sections of the brains were stained with 0.25% cresyl violet (Sigma-Aldrich, USA), dehydrated through graded alcohols (70, 95, 100% 2x) (RCI LabScan, Thailand), placed in xylene (Merck, Germany), and mounted using DPX mountant (Merck, Germany). The evaluation of neuron density in the hippocampus was performed under Olympus light microscope model BH-2 (Japan) at 40x magnification. Counts were performed in three adjacent fields, and the mean number was calculated and expressed as the density of neurons per 255 *μ*m^2^.

### 2.11. Assessment of Oxidative Stress Status and Acetylcholine Activity in Hippocampus

At the end of the study, all animals were sacrificed. Anterior hippocampus was isolated, prepared as hippocampal homogenate with 50 volume of 0.1 M phosphate buffer saline. Then, the homogenate was used for the determination of the acetylcholinesterase (AChE) activity and oxidative status including malondialdehyde (MDA) level and the activities of superoxide dismutase (SOD), catalase (CAT), and glutathione peroxidase (GSH-Px). The protein concentration in brain homogenate was determined by using a Thermo Scientific NanoDrop 2000c spectrophotometer (Thermo Fisher Scientific, Wilmington, DE, USA) and measured the optical density at the wavelength of 280 nm.

The determination of AChE in brain homogenate was evaluated according to the spectrophotometric method of Elmann et al. with a slight modification. [19]. The mixture of 20 *μ*l of sample solution, 200 *μ*l of 0.1 mM sodium phosphate buffer (pH 8.0) (Sigma-Aldrich, USA), and 10 *μ*l of 0.2 M DTNB (5,5′-dithio-bis-(2-nitrobenzoic acid)) (Sigma-Aldrich, USA) were mixed and incubated at room temperature for 5 minutes. Then, 10 *μ*l of 15 mM acetylcholine thiochloride (ACTI) (Sigma-Aldrich, USA) was added and incubated for 3 minutes and recorded the absorbance at 412 nm by using microplate reader (iMark Microplate Absorbance Reader). The activity of AChE was calculated according to the equation below and expressed as nmol/min·mg protein.

(1) $$\begin{array}{c} AChE\;activity=\frac{\Delta A}{1.36\times 10^{4}}\times \frac{1}{20/230}C, \end{array}$$

where ∆*A* = the difference of absorbance/minute and C = protein concentration of brain homogenate.

The level of MDA level, a lipid peroxidation product, was also assessed. Hippocampal homogenate was determined by thiobarbituric acid reaction according to the method of Ohkawa et al. [20, 21]. The reaction mixture containing 50 *μ*l of sample solution, 50 *μ*l of 8.1% sodium dodecyl sulfate (SDS) (Sigma-Aldrich, USA), 375 *μ*l of 0.8% of thiobarbituric acid (TBA) (Sigma-Aldrich, USA), 375 *μ*l of 20% acetic acid (Sigma-Aldrich, USA), and 150 *μ*l of distilled water (DW) was boiled at 95°C in the water bath for 60 minutes. After boiling, it was cooled with tap water. Then, 250 *μ*l of DW and 1250 *μ*l of the solution containing n-butanol and pyridine (Merck, Germany) at the ratio of 15 : 1 were added, mixed together, and centrifuged at 4000 rpm for 10 minutes. The upper layer was separated and measured the absorbance at 532 nm. 1,1,3,3-tetramethoxy propane (0–15 *μ*M) (Sigma-Aldrich, USA) was served as standard, and the level of MDA was expressed as ng/mg protein.

The determination of SOD activity was performed according to the method of Sun et al. [22]. In brief, the assay mixture containing 57 mM phosphate buffer solution (KH_2_PO_4_) (Sigma-Aldrich, USA), 0.1 mM EDTA (Sigma-Aldrich, USA), 10 mM cytochrome C (Sigma-Aldrich, USA) solution, and 50 *μ*M of xanthine (Sigma-Aldrich, USA) solution at the volume of 200 *μ*l was mixed with 20 *μ*l of tissue sample. Then, 20 *μ*l of xanthine oxidase (0.90 mU/ml) (Sigma-Aldrich, USA) solution was added and the absorbance at 415 nm was measured. SOD enzyme (Sigma-Aldrich, USA) activities at the concentrations of 0–25 units/ml were used as standard, and the results were expressed as units/mg protein.

Catalase activity was determined based on the ability of the enzyme to break down H_2_O_2_. In brief, 10 *μ*l of sample was mixed with the reaction mixture containing 50 *μ*l of 30 mM hydrogen peroxide (in 50 mM phosphate buffer, pH 7.0) (BDH Chemicals Ltd, UK), 25 *μ*l of H_2_SO_4_ (Sigma-Aldrich, USA), and 150 *μ*l of 5 mM KMnO_4_ (Sigma-Aldrich, USA). After mixing thoroughly, the optical density was measured at 490 nm [23]. CAT enzyme (Sigma-Aldrich, USA) at the concentration range of 0–100 units/ml was used as standard, and the result was expressed as units/mg protein.

Glutathione peroxidase activity was also assessed. In brief, a mixture containing a 20 *μ*l of sample solution and the reaction mixture consisting of 10 *μ*l of 1 mM dithiothreitol (DTT) (Sigma-Aldrich, USA) in 6.67 mM potassium phosphate buffer (pH 7), 100 *μ*l of 1 mM sodium azide (Sigma-Aldrich, USA) in 6.67 mM potassium phosphate buffer (pH 7), 10 *μ*l of 50 mM glutathione (Sigma-Aldrich, USA) solution, and 100 *μ*l of 30% hydrogen peroxide (BDH Chemicals Ltd, UK) were mixed thoroughly and incubated at room temperature for 5 minutes. Then, 10 *μ*l of 10 mM DTNB (5,5-dithiobis-2-nitrobenzoic acid) (Sigma-Aldrich, USA) was added and the optical density at 412 nm was recorded at 25°C over a period of 5 min [24]. The standard calibration curve was prepared by using GSH-Px enzyme (Sigma-Aldrich, USA) at the concentration range of 0–5 units/ml. GSH-Px activity was expressed as units/mg protein.

### 2.12. Western Blotting Analysis

Anterior hippocampus of the brain was suspended and homogenized in mammalian protein extraction reagent (M-PER; Pierce Protein Biology Product, Rockford, IL, USA), with protease inhibitor cocktail (1 : 10) (Sigma-Aldrich, USA). Brain homogenate was subjected to a 12,000*g* centrifugation process for 10 minutes at 4°C. The supernatant was isolated and used for the determination of protein and Erk expression. Protein concentration was determined by using a Thermo Scientific NanoDrop 2000c spectrophotometer (Thermo Fisher Scientific, Wilmington, DE, USA). In addition, sixty micrograms of tissue lysates was adjusted to an appropriate concentration by using Tris-Glycine SDS-PAGE loading buffer (Bio-Rad, USA) and heated at 95°C for 10 minutes. Protein in tissue sample was isolated via sodium dodecyl sulfate-polyacrylamide gel electrophoresis (SDS-PAGE) by loading 20 *μ*l of tissue sample on SDS-polyacrylamide gel. Then, the separated bands were transferred to nitrocellulose membrane, washed with 0.05% TBS-T, and incubated in blocking buffer (5% skim milk in 0.1% TBS-T) at room temperature for 1 hour. After the blocking process, the nitrocellulose membrane was incubated with anti-phospho-Erk1/2 (Thr202/Tyr204) (Cell Signaling Technology, USA; dilution 1 : 1000), anti-Erk1/2 (Cell Signaling Technology, USA; dilution 1 : 1000) antibodies at room temperature for 2 hours. The nitrocellulose membrane was rinsed with TBS-T (0.05%) again and incubated with anti-rabbit IgG, HRP-linked antibody (Cell Signaling Technology, USA; dilution 1 : 2000) at room temperature for 1 hour. The bands were visualized and quantitated by using the ECL detection systems (GE Healthcare) and LAS-4000 luminescent image analyzer (GE Healthcare). Band intensities were measured for statistical analysis using ImageQuant TL v.7.0 image analysis software (GE Healthcare). The expression was normalized using anti-total ERK1/2. Data were presented as a relative density to control normal diet group.

### 2.13. Statistical Analysis

All data are expressed as mean ± standard error of mean (SEM). Statistical significance was evaluated by using one-way analysis of variance (ANOVA), followed by Duncan's post hoc test. The statistical significance was regarded at *p* values < 0.05.

## 3. Results

### 3.1. Assessment of Bioactive Compounds and Biological Activities

Table 1 showed that the contents of polyphenolic compounds and flavonoids in mulberry fruit extract were 80 ± 0.98 mg of gallic acid equivalent (GAE)/mg sample extract and 8.89 ± 0.13 *μ*g quercetin/mg sample extract whereas the contents of the substances just mentioned in microencapsulated mulberry fruit extract (MME) were 103.89 ± 13.08 mg GAE/mg sample extract and 26.56 ± 1.26 *μ*g quercetin/mg sample extract. The flavonoid content in MME is significantly higher than that in mulberry fruit extract (*p* value < 0.01, compared between MME and mulberry fruit extract). Half maximal effective concentration (EC_50_) of antioxidant evaluated via DPPH, FRAP, and ABTS assays of mulberry fruit extract was 2.56 ± 0.08, 280.04 ± 3.14, and 4.08 ± 0.02 mg/ml, respectively. EC_50_ of antioxidant activity of MME assessed by DPPH, FRAP, and ABTS were 2.07 ± 0.79, 192.79 ± 15.94, and 1.66 ± 0.08 mg/ml. Although MME shows the potent antioxidant activity than mulberry extract, the significant difference was observed only the EC_50_ assessed via ABTS assay (*p* value < 0.01, compared between MME and mulberry fruit extract). In addition, the EC_50_ of antioxidant effect of MME assessed via the mentioned methods was very much higher than the reference. EC_50_ of AChEI activity of a mulberry fruit extract was 0.07 ± 0.002 while that of MME was 0.05 ± 0.003 mg/ml. Interestingly, EC_50_ of AChEI of MME was significantly lower than that of mulberry extract (*p* value < 0.05, compared between MME and mulberry fruit extract). In addition, this value is close to the value of donepezil, a standard drug used for treating dementia.

### 3.2. The Finger Print of Microencapsulated Mulberry Fruit Extract

**Figure 1:**
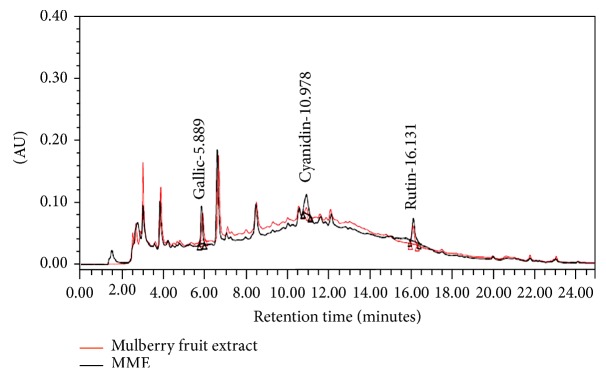
The fingerprint chromatogram of mulberry fruit extract and microencapsulated mulberry fruit extract (MME).

Figure 1 shows the fingerprint chromatogram of mulberry fruit extract and microencapsulated mulberry fruit extract (MME). The fingerprint chromatogram analysis obtained from this study revealed that mulberry fruit extract of 200 milligrams contained 253.04 ± 3.92 *μ*g cyanidin-3-glucoside, 10.81 ± 0.29 *μ*g gallic acid, and 265.84 ± 17.66 *μ*g quercetin-3-O-rutinoside whereas MME of 200 milligrams contained of 293.62 ± 4.90 *μ*g cyanidin-3-glucoside, 9.08 ± 0.09 *μ*g gallic acid, and 243.51 ± 5.88 *μ*g quercetin-3-O-rutinoside. However, no significant difference in gallic acid and quercetin-3-O-rutinoside contents between MME and mulberry fruit extract was observed (*p* value > 0.05). Interestingly, the cyanidin-3-glucoside content in MME is significantly higher than that in mulberry fruit extract (*p* value < 0.05, compared between MME and mulberry fruit extract).

### 3.3. Effect of MME on Spatial Memory

**Figure 2:**
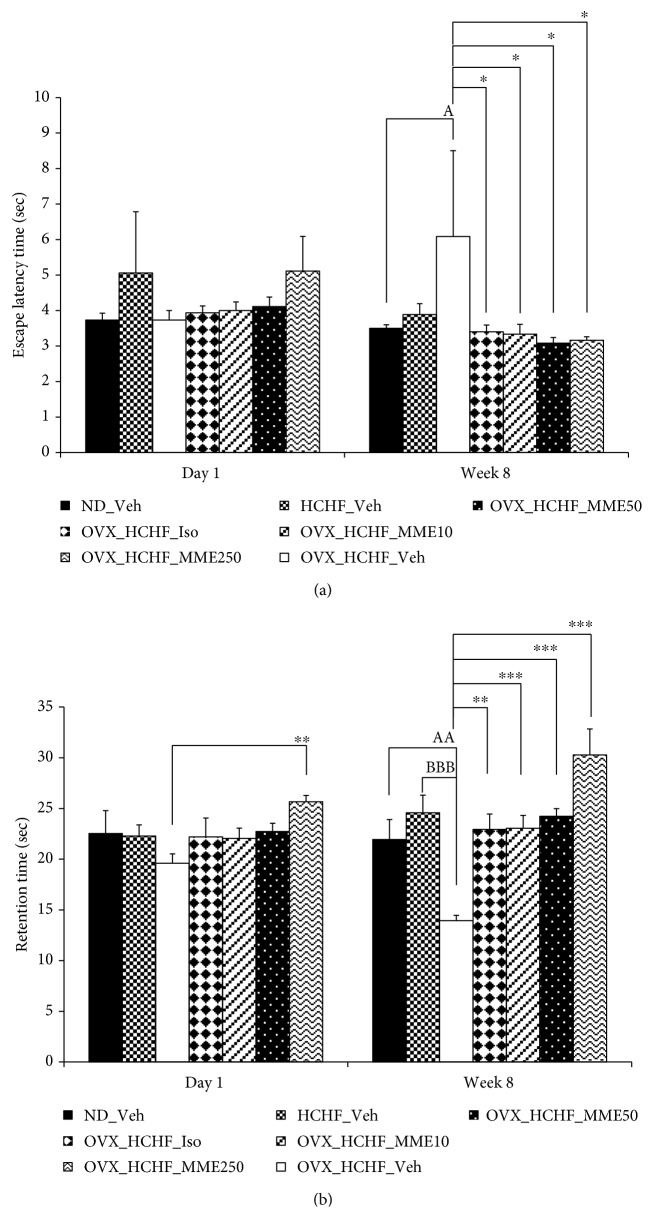
Effect of microencapsulated mulberry fruit extract (MME) on memory impairment via Morris Water Maze test ((a) escape latency time, (b) retention time). Data are presented as mean ± SEM (*n* = 6/group). ^a^*p* value < 0.05 and ^aa^*p* value < 0.01, compared to control rats which received normal diet and vehicle; ^bbb^*p* value < 0.001, compared to normal rats which received HCHF diet and vehicle; and ^∗^*p* value < 0.05, ^∗∗^*p* value < 0.01, ^∗∗∗^*p* value < 0.001, compared to OVX rats which received HCHF and vehicle. ND: normal diet; HCHF: high-carbohydrate high-fat diet; OVX-HCHF: ovariectomized plus high-carbohydrate high-fat diet; Iso: the isoflavone at dose of 15 mg·kg^−1^ BW; MME10, 50, and 250: the microencapsulated mulberry fruit extract at dose of 10, 50, and 250 mg·kg^−1^ BW, respectively.

The effect of MME on escape latency was shown in Figure 2(a). After the single administration, no significant differences among groups were observed. When compared between rats which received HCHF diet and those which received normal diet, no significant change in escape latency was observed after 8 weeks of treatment. However, HCHF diet induced the increased escape latency in OVX rats (*p* value < 0.05, compared to control rats which received normal diet and vehicle). In addition, OVX rats which received HCHF diet and received isoflavone or received MME at doses of 10, 50, and 250 mg/kg significantly decreased escape latency (*p* value < 0.05 all, compared to OVX rats which received HCHF diet and vehicle).

Figure 2(b) shows the effect of MME on retention time. After the single administration, HCHF diet failed to produce the significant changes of retention time in both normal and OVX rats. OVX rats which received a high dose of MME significantly increased retention time after the single administration (*p* value < 0.01, compared to OVX rats which received HCHF and vehicle). After 8 weeks of intervention, it was found that HCHF failed to produce the significant change in retention time in normal rats which received vehicle. However, OVX rats which received HCHF diet significantly decreased retention time (*p* value < 0.01, compared to normal rats which received vehicle; *p* value < 0.001, compared to normal rats which received HCHF diet). Both OVX rats which fed with HCHF diet and received either isoflavone or MME (all doses used in this study) significantly enhanced retention time (*p* value < 0.01, 0.001, 0.001, and 0.001, respectively, compared to OVX which received HCHF diet and vehicle). Interestingly, MME at a dose of 250 mg/kg also enhanced retention time better than normal rats which received vehicle.

### 3.4. Effect of MME on Hippocampal Neuron

**Figure 3:**
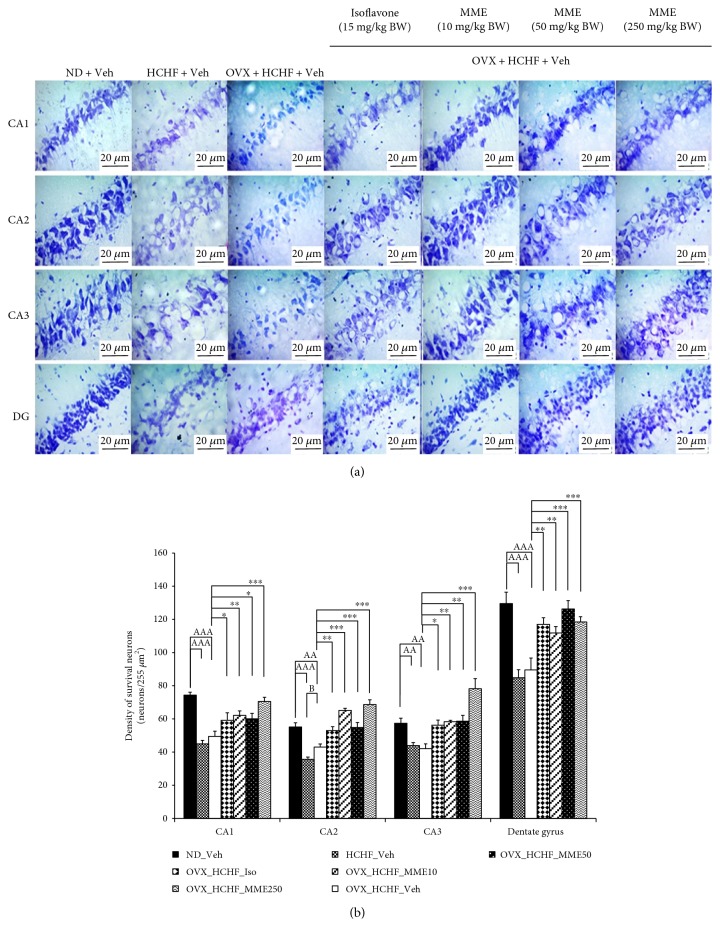
Effect of microencapsulated mulberry fruit extract on density of survival neurons in the various subregions of the hippocampus. (a) Light microscope of coronal sections in CA1, CA2, CA3, and dentate gyrus of the hippocampus were stained with cresyl violet at 40x magnification. (b) Density of survival neurons in CA1, CA2, CA3, and dentate gyrus of the hippocampus. Data are presented as mean ± SEM (*n* = 6/group). ^aa^*p* value < 0.01 and ^aaa^*p* value < 0.001, compared to control rats which received normal diet and vehicle; ^b^*p* value < 0.05, compared to normal rats which received HCHF diet and vehicle; and ^∗^*p* value < 0.05, ^∗∗^*p* value < 0.01, ^∗∗∗^*p* value < 0.001, compared to OVX rats which received HCHF and vehicle. ND: normal diet; HCHF: high-carbohydrate high-fat diet; OVX-HCHF: ovariectomized plus high-carbohydrate high-fat diet; Iso: the isoflavone at dose of 15 mg·kg^−1^ BW; MME10, 50, and 250: the microencapsulated mulberry fruit extract at dose of 10, 50, and 250 mg·kg^−1^ BW, respectively.

To further explore the possible underlying mechanism of the memory-enhancing effect of MME, the plasticity of the hippocampus was assessed via the assessment of the neuron density in CA1, CA2, CA3, and the dentate gyrus of the hippocampus (Figure 3). It was found that normal rats which received HCHF diet significantly decreased neuron density in CA1, CA2, CA3, and dentate gyrus (*p* value < 0.001, 0.001, 0.01, and 0.001, respectively, compared to normal rats which received normal diet and vehicle). OVX rats which received HCHF diet also showed the reduction of neuron density in all subregions mentioned earlier (*p* value < 0.001, 0.01, 0.01, and 0.001, respectively, compared to normal rats which received normal diet and vehicle). HCHF diet decreased the neuron density in CA2 of OVX rats higher than normal rats (*p* value < 0.05, compared between OVX rats which received HCHF diet and normal rats which received HCHF diet). Isoflavone could counteract the reduction of neuron density in CA1, CA2, CA3, and dentate gyrus in OVX rats which fed with HCHF diet (*p* value < 0.05, 0.01, 0.05, and 0.01, respectively, compared to OVX rats which received HCHF diet and vehicle). MME at all doses used in this study also enhanced neuron density in all subregions just mentioned in OVX rats which received HCHF diet (*p* value < 0.01, 0.05, and 0.001; *p* value < 0.001 all; *p* value < 0.01, 0.01, and 0.001; 0.01, 0.001, and 0.001, respectively, compared to OVX rats which received HCHF diet and vehicle). Interestingly, the increased neuron density in CA2 and CA3 induced by high dose of MME and the increased neuron density in CA2 induced by the low dose of MME were higher than the neuron density in the normal healthy rats which received normal diet and vehicle.

### 3.5. Effect of MME on Oxidative Stress Status and AChE in the Hippocampus

Table 2 shows the effect of MME on AChE activity and oxidative stress markers including MDA level and the activities of SOD, CAT, and GSH-Px. In this study, the effect of MME on AChE was assessed in order to indirectly indicate the effect on the available acetylcholine (ACh) and cholinergic function. The current data showed that HCHF diet failed to produce the significant change of AChE in normal rats but it significantly increased AChE activity in the hippocampus of OVX rats (*p* value < 0.001, compared to normal rats which received normal diet and vehicle; *p* value < 0.001, compared to normal rats which received HCHF diet and vehicle). OVX rats which received HCHF diet and isoflavone significantly decreased AChE activity in the hippocampus (*p* value < 0.01, compared to OVX rats which received HCHF diet and vehicle). The reduction of AChE in this area was also observed in OVX rats which received MME at all doses used in this study (*p* value < 0.01, 0.01, and 0.05, respectively, compared to OVX rats which received HCHF diet and vehicle). The decreased activities of SOD, CAT, and GSH-Px (*p* value < 0.001 all, compared to normal rats which received normal diet and vehicle; *p* value < 0.001 all, compared to normal rats which received HCHF diet and vehicle) together with the increased MDA level (*p* value < 0.01, compared to normal rats which received normal diet) were also observed in OVX rats which received HCHF diet. Isoflavone treatment significantly increased SOD and CAT activities but decreased MDA level in OVX rats which received HCHF (*p* value < 0.05 all, compared to OVX rats which received HCHF diet and vehicle). MME at all doses used in this study also increased SOD (*p* value < 0.001, 0.05, and 0.01, respectively, compared to OVX rats which received HCHF diet and vehicle), CAT (*p* value < 0.01 all, compared to OVX rats which received HCHF diet and vehicle), and GSH-Px (*p* value < 0.01, 0.05, and 0.01, respectively, compared to OVX rats which received HCHF diet and vehicle) but decreased MDA level (*p* value < 0.05, 0.01, and 0.01, respectively, compared to OVX rats which received HCHF diet and vehicle) in hippocampus.

### 3.6. Effect of MME on the Phosphorylation of Erk in the Hippocampus

**Figure 4:**
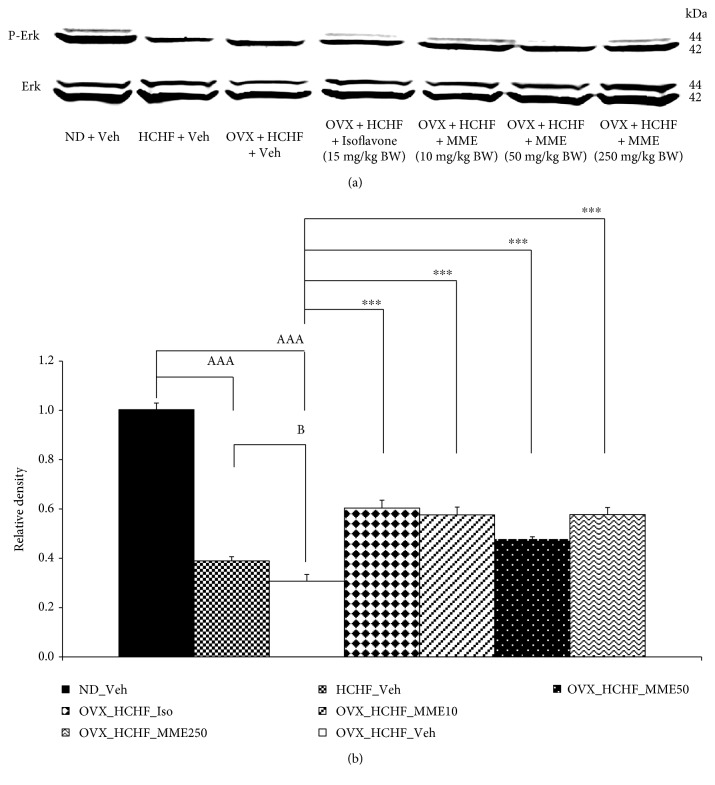
Effect of various doses of microencapsulated mulberry fruit extract on the relative density of total P-Erk/Erk in the hippocampus was detected by Western blotting (a) and quantitatively analysis (b). The levels of total P-Erk were normalized against the level of total Erk. Their relative phosphorylation levels (active form) were calculated against those of control normal diet plus vehicle rats. Data are presented as mean ± SEM (*n* = 6/group). ^aaa^*p* value < 0.001, compared to control rats which received normal diet and vehicle, ^b^*p* value < 0.05, compared to normal rats which received HCHF diet and vehicle, and ^∗∗∗^*p* value < 0.001, compared to OVX rats which received HCHF and vehicle. ND: normal diet; HCHF: high-carbohydrate high-fat diet; OVX-HCHF: ovariectomized plus high-carbohydrate high-fat diet; MME10, 50, and 250: the microencapsulated mulberry fruit extract at dose of 10, 50, and 250 mg·kg^−1^ BW, respectively.

Figure 4 shows the effect of MME on the phosphorylation of Erk in the hippocampus. It was found that HCHF diet significantly decreased the phosphorylation of Erk in the hippocampus of both normal rats and OVX rats (*p* value < 0.001, compared to normal rats which received normal diet and vehicle). However, the magnitude of change in OVX rats was higher than that in normal rats (*p* value < 0.05, compared between normal rats which received HCHF diet and vehicle and OVX rats which received HCHF diet and vehicle). In addition, isoflavone and all doses of MME could counteract the reduction of Erk phosphorylation induced by HCHF diet in OVX rats (*p* value < 0.001, compared to OVX rats which received HCHF diet and vehicle).

## 4. Discussion

The present study has demonstrated that encapsulated mulberry fruit extract contains more polyphenolic compounds and flavonoid content. In addition, the biological activity including antioxidant and AChEI activities of the encapsulated mulberry fruit extract is also better than that of the mulberry fruit extract. In this study, both the mulberry fruit extract and encapsulated mulberry fruit extract were determined the contents of phenolic compounds and flavonoids 2 weeks after the preparation. Based on the antioxidant and AChEI of polyphenol [25, 26], the loss of some phenolic compounds and flavonoid contents during storage may possibly involve with the different contents of both substances mentioned earlier. The loss of phenolic compounds and flavonoid contents of mulberry fruit extract during storage due to environmental factors such as light and temperature [27, 28] may probably be more than the losses of the mentioned substances of encapsulated mulberry fruit giving rise to the higher contents of both substances in encapsulated mulberry fruit extract than that in mulberry fruit extract.

Our data also clearly demonstrates that the encapsulated mulberry fruit extract significantly improves spatial memory in an animal model of menopause with metabolic syndrome. Interestingly, the high dose of encapsulated mulberry fruit extract can significantly enhance retention time after the single administration while isoflavone fails to show the positive modulation effect on this parameter after the single-dose administration. However, at the end of the 8-week intervention period, isoflavone and all doses of encapsulated mulberry fruit extract used in this study show the positive modulation effect on spatial memory. Since the hippocampus is the brain area that plays an important role in spatial memory [29, 30], the changes of parameters contributing the important roles on learning and memory such as ACh [31] and oxidative stress [32] were also investigated in this area. It has been found that in accompany with the improved spatial memory, the decreased AChE and MDA level together with the elevations of SOD, CAT, and GSH-Px were also present. Accumulative lines of evidence have shown that oxidative stress status in the hippocampus plays a pivotal role on the neuron survival in the hippocampus and memory performance [10, 29, 30]. Therefore, the improved learning and memory impairment observed in this study may occur partly via the improved function of cholinergic system via the increase of ACh and the decreased oxidative stress status in the hippocampus.

A pile of evidence has demonstrated that the brain plasticity which contributes a crucial role on learning and memory is associated with the neuron density in the hippocampus area [10, 33]. Therefore, the effects of encapsulated mulberry fruit extract on neuron densities in CA1, CA2, CA3, and dentate gyrus of the hippocampus were also explored. Interestingly, all doses of encapsulated mulberry fruit extract enhanced neuron density in all areas mentioned earlier. It has been reported that the phosphorylation of extracellular signal-regulated kinase (ERK) also plays an important role on the neuroprotection and brain plasticity [34] so we also investigated the effect of encapsulated mulberry fruit extract on the phosphorylation of Erk. Our data have revealed that encapsulated mulberry fruit extract-treated rats also increased the phosphorylation of Erk. Taken all data together, we suggested that the increased brain plasticity which occurred via the increased phosphorylation of Erk might also play a role on the memory-enhancing effect of encapsulated mulberry fruit extract. Since, the increased neuron densities induced by MME at low and high doses of MME in CA2 and CA3 are higher than those in normal rats, we suggest that MME at the mentioned dose might also increase neurogenesis in the mentioned areas. However, further exploration is required to confirm this event.

No dose-dependent responses were observed in this study because the memory performance depended on many factors, so no simple linear relationship between the concentrations of the tested substance and the observed parameters was observed. In addition, the tested substance contained numerous ingredients and the effect of active ingredient can be masked by the others.

Based on the high contents of phenolic compounds such as cyanidin-3-glucoside, gallic acid, and quercetin-3-O-rutinoside in microencapsulated mulberry fruit extract together with the neuroprotection and memory-enhancing effects of the substances just mentioned [35–39], the neuroprotection and memory-enhancing effects of microencapsulated mulberry fruit extract observed in this study might be related to the contents of aforementioned phenolic compounds. However, the modulation effect induced by the interaction effects among various ingredients may also play the roles. Therefore, the precise understanding concerning this aspect required further study.

**Figure 5:**
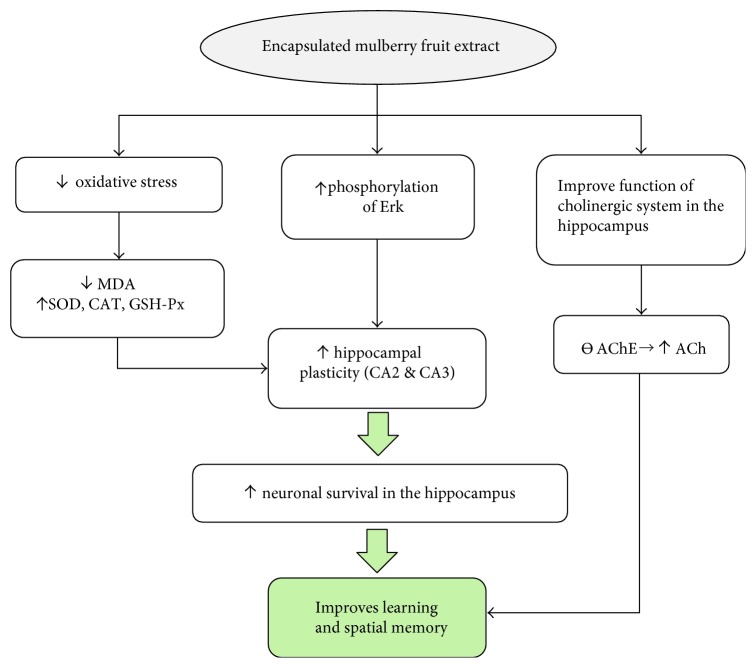
The possible underlying mechanism for the neuroprotective and cognitive-enhancing effect of encapsulated mulberry fruit extract on an animal model of menopause with metabolic syndrome.

In summary, Figure 5 shows that MME exerts the memory-enhancing effect via multiple targets including the improved cholinergic function and the brain plasticity. MME can suppress AChE in the hippocampus which in turn improves cholinergic function in this area resulting in the improved memory performance. Moreover, MME also enhances plasticity of the hippocampus via both the decreased oxidative stress status and the increased Erk phosphorylation and finally giving rise to the improved memory. Since the low and high doses of MME can also increase neuron density in CA2 and CA3 than that in normal rats, MME at the doses mentioned earlier may probably increase neurogenesis in both subregions mentioned earlier. However, this point also requires further exploration.

## 5. Conclusion

Encapsulated mulberry fruit extract shows higher stability of phenolic compounds including flavonoids giving rise to the potent antioxidant and AChEI. The current preclinical data also clearly show that it improves hippocampal plasticity and memory impairment in an animal model of experimental menopause with metabolic syndrome. Therefore, it may be served as the potential candidate for neuroprotectant and memory enhancer for menopausal women with metabolic syndrome. However, further researches concerning the possible active ingredients, the effect on neurogenesis, and clinical trial are still essential.

## Figures and Tables

**Table 1:** The bioactive compounds and biological activities of microencapsulated mulberry fruit extract (MME).

Parameters	Mulberry fruit extract	Microencapsulated mulberry fruit extract	Standard reference
Total phenolic compounds (mg GAE/mg extract)	80.00 ± 0.98	103.89 ± 13.08	—
Flavonoids content (*μ*g quercetin/mg extract)	8.89 ± 0.13	26.56 ± 1.26^∗∗^	—
DPPH (EC_50_, mg/ml)	2.56 ± 0.08	2.07 ± 0.79	0.03 ± 0.01, ascorbic acid
FRAP (EC_50_, mg/ml)	280.04 ± 3.14	192.79 ± 15.94	21.04 ± 1.24, ascorbic acid
ABTS (EC_50_, mg/ml)	4.08 ± 0.02	1.66 ± 0.08^∗∗^	0.20 ± 0.002, Trolox
AChEI (EC_50_, mg/ml)	0.07 ± 0.002	0.05 ± 0.003^∗^	0.02 ± 0.001, donepezil

Data are presented as mean ± SEM. ^∗^*p* value <0.05 and ^∗∗^*p* value < 0.01, compared between MME and mulberry fruit extract.

**Table 2:** The effect of various doses of MME on acetylcholineseterase activity and oxidative stress markers in the hippocampus.

Treatment group	AChE activity (nmol/min·mg protein)	MDA (ng/mg protein)	SOD (units/mg protein)	CAT (units/mg protein)	GSH-Px (units/mg protein)
ND + vehicle	0.14 ± 0.01	4.38 ± 1.41	59.28 ± 6.40	780.07 ± 104.63	10.86 ± 1.04
HCHF + vehicle	0.17 ± 0.04	14.27 ± 2.36^aaa^	47.39 ± 5.86^a^	517.12 ± 53.99^aa^	7.29 ± 0.96^aa^
OVX + HCHF + vehicle	0.43 ± 0.12^aaa,bbb^	11.85 ± 0.85^aa^	14.28 ± 0.82^aaa,bbb^	201.80 ± 12.04^aaa,bbb^	3.64 ± 0.52^aaa,bbb^
OVX + HCHF + isoflavone 15 mg/kg BW	0.19 ± 0.03^∗∗^	7.05 ± 0.69^∗^	30.74 ± 2.71^∗^	411.66 ± 37.81^∗^	4.72 ± 0.42
OVX + HCHF + MME 10 mg/kg BW	0.20 ± 0.03^∗∗^	6.51 ± 0.98^∗^	38.15 ± 2.53^∗∗∗^	446.31 ± 51.80^∗∗^	6.94 ± 0.69^∗∗^
OVX + HCHF + MME 50 mg/kg BW	0.20 ± 0.03^∗∗^	4.93 ± 0.61^∗∗^	30.07 ± 0.56^∗^	427.58 ± 15.64^∗∗^	6.06 ± 0.36^∗^
OVX + HCHF + MME 250 mg/kg BW	0.26 ± 0.02^∗^	5.06 ± 0.69^∗∗^	31.40 ± 1.64^∗∗^	464.92 ± 38.16^∗∗^	7.27 ± 0.43^∗∗^

Data are presented as mean ± SEM (*n* = 6/group). ^a^*p* value < 0.05, ^aa^*p* value < 0.01, ^aaa^*p* value < 0.001, compared to control rats which received normal diet and vehicle, ^bbb^*p* value < 0.001, compared to normal rats which received HCHF diet and vehicle and ^∗^*p* value < 0.05, ^∗∗^*p* value < 0.01, ^∗∗∗^*p* value < 0.001, compared to OVX rats which received HCHF and vehicle.
